# Communication between Plants and Rhizosphere Microbiome: Exploring the Root Microbiome for Sustainable Agriculture

**DOI:** 10.3390/microorganisms11082003

**Published:** 2023-08-03

**Authors:** Ben Jesuorsemwen Enagbonma, Ayomide Emmanuel Fadiji, Ayansina Segun Ayangbenro, Olubukola Oluranti Babalola

**Affiliations:** Food Security and Safety Focus Area, Faculty of Natural and Agricultural Sciences, North-West University, Private Mail Bag X2046, Mmabatho 2735, South Africa

**Keywords:** plant-microbe interactions, signaling molecule, root exudate, disease suppression, crop production

## Abstract

Plant roots host numerous microorganisms around and inside their roots, forming a community known as the root microbiome. An increasing bulk of research is underlining the influences root-associated microbial communities can have on plant health and development. However, knowledge on how plant roots and their associated microbes interact to bring about crop growth and yield is limited. Here, we presented (i) the communication strategies between plant roots and root-associated microbes and (ii) the applications of plant root-associated microbes in enhancing plant growth and yield. This review has been divided into three main sections: communications between root microbiome and plant root; the mechanism employed by root-associated microbes; and the chemical communication mechanisms between plants and microbes and their application in plant growth and yield. Understanding how plant root and root-associated microbes communicate is vital in designing ecofriendly strategies for targeted disease suppression and improved plant growth that will help in sustainable agriculture. Ensuring that plants become healthy and productive entails keeping plants under surveillance around the roots to recognize disease-causing microbes and similarly exploit the services of beneficial microorganisms in nutrient acquisition, stress mitigation, and growth promotion.

## 1. Introduction

The orthodox methods of attaining improved agricultural output are not environmentally sustainable. For instance, the superfluous and prolonged application of artificial agrochemicals results in soil nutrients depletion and water pollution as well as environmental degradation [1]. If nothing is done to mitigate the effect of the excessive and prolonged use of agrochemicals, coupled with the human population predicted to hit 9.9 billion by 2030, there will be a decline in the amount of food resources available to feed the world [2]. Hence, sustainable agricultural practices like the use of biological materials is a necessity for restoring soil fertility, feeding the ever-increasing population, and improving the agroecosystem resilience [3].

**Figure 1:**
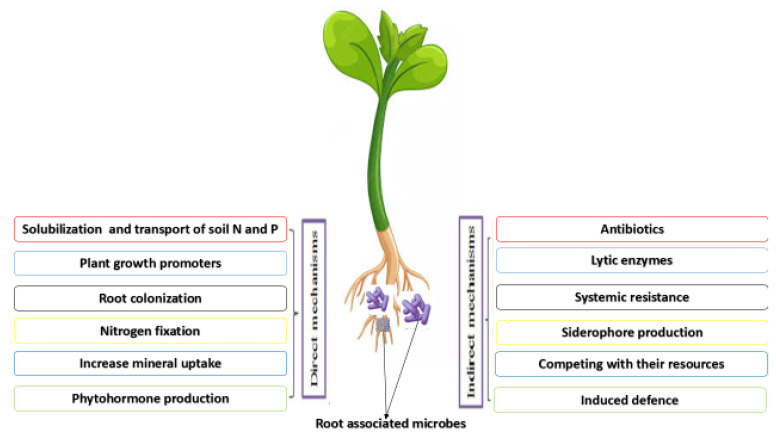
Mechanisms employed by plant root-associated microbes in improving plant health.

**Figure 2:**
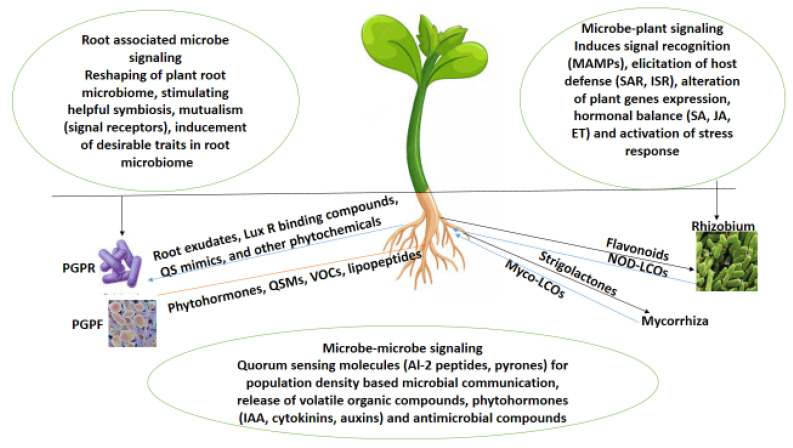
Plant root-associated microbe communication.

Plant microbiome nexuses could provide a prospect to develop schemes for sustainable agricultural practices [4]. Plants take up water and nutrients through the root system, which is inhabited and bounded by a multifaceted microbial community referred to as the root microbiome [5]. The root microbiome is a significant driver for plant yield, health, and ecosystem functioning because it is the intersection point between a plant and the ecosphere [6]. Furthermore, it serves as a receptacle of extra genes that plants can acquire when required [7]. The root microorganisms are recruited from several microorganisms found in bulk soil, which is the basic factor influencing the composition of the root microbiome [8]. It is also vital to note that the plant genotype also contributes to the final composition of these societies because plant-resultant substrates and exudates give the nutrient and physical niches of the rhizosphere [9]. These microorganisms form complex links that are established and controlled via antagonism, competition, nutrient cycling, and chemical communication facilitated by various groups of signaling molecules (Figure 1 and Figure 2) [10].

Understanding the concept and relevance of the root microbiome to plant health will improve our insight into the colossal power of these tiny giants in ecosystem function. In recent years, research has focused on the composition and the structure of root microorganisms [11]. However, the multifarious communications between the root microbiome and the host are not entirely understood. These interactions could facilitate the release of plant growth regulators; nitrogen fixation; zinc, potassium, and phosphate solubilization; siderophore, hydrogen cyanide, and ammonia production; and the production of other secondary metabolites that are hostile to disease-causing organisms [12]. With this in mind, the study aimed to review (i) the communication mechanisms between plant roots and its associated plant growth-promoting microbial communities and (ii) the applications of plant root-associated microbes in enhancing plant growth and yield. This understanding will provide sustainable solutions in raising agronomic crop production. This review was sectioned into communications between the root microbiome and plant root; the mechanism employed by root-associated microbes in promoting plant growth; and the chemical communication mechanisms between plants and microbes and their application in plant growth and yield through stress mitigation, disease suppression, and nutrient acquisition.

## 2. Communication between the Root Microbiome and Plant Root

The communication between the root microbiome and plant roots is an intricate and dynamic process that entails an array of chemical, molecular, and physical interactions [13]. These interactions are controlled by a wide range of specialized exudates and metabolites [14]. Around or within the plant roots, there are several of these molecules whose concentration varies according to the distance from the point of emission. These deposits include sloughed-off tissue and cells, H^+^ efflux, CO_2_ from cell respiration, mucilage, intact root border cells, and proteins [8]. The low molecular weight organic compounds, also known as root exudates, contain amides, sugars, and phenolic, aromatic, and amino acids [15]. These chemicals facilitate communications, function as chemical attractants and repellants that drive the root microbiome, and include bacteria, fungi, archaea, and viruses that reside in and around the roots of plants [15].

The underlying interaction among bacterial microbes is unquestionably a vital factor in root microbiome dynamics [16]. This interaction between bacterial cells is dependent on the production and distribution of signal molecules that is consequently perceived by other community affiliates. Upon signal recognition, the molecules can be up- or down-regulating gene expression and alter the physiology and activities of the receiving organism [17,18]. These communications exert both negative and positive impacts on the agricultural scheme based on the relationship (whether pathogenic, symbiotic, or growth promoting) that occurs [19]. Fungi, on the other hand, use their arm-like and branching membrane to form a mycelium, a communication system that links between plant roots [20]. The mycelium provide water, sugar, and nutrients, and in a more intricate dynamics with the plants, provide chemical signals. With plant root-fungi interaction, plants can indirectly communicate with other plants around them [21]. The indirect communication depends on the fungal network, which allows the flow of various chemical signals [22]. For example, a high level of soil phosphorus tells other plants that there is a plant-fungal collaboration, and they may reply to this indicator either by producing sugar in order to entice these kinds of fungi so that they can obtain their share of nutrients or by releasing chemicals to wane the fungus’ capacities to manufacture nutrients, thereby rendering their competitor less healthy [23]. Archaea directly relate with plants, and they have the potential to communicate with plants through (i) nutrient supply, (ii) possible plant growth promotion via auxin biosynthesis, and (iii) fortification against abiotic (mainly osmotic and oxidative) stress [24,25].

The communication between the root microbiome and plant roots involves a sophisticated interplay of chemical signals, root exudates, microbial metabolites, and gene regulation. This dynamic interaction is crucial for plant growth, health, and adaptation to changing environmental conditions [26]. For instance, the root-associated microbes employ direct or indirect strategies to impact plant health status and growth [27]. Direct strategies include nutrient acquisition, phytohormones production, and phosphate solubilization, while indirect strategies include eliciting plant immune responses and preventing plant pathogens from proliferating and competing with their resources (Figure 1) [28]. For example, Stringlis, et al. [29] showed that probiotic-plant-rhizobacteria collaboration elicited the root-specific transcription factor MYB72 and further led to the production and emission of MYB72-controlled β-glucosidase BGLU42-reliant scopolin and scopoletin, respectively, resulting in a well-established niche for microbial consortiums and resistance profits for the host plant against *Verticillium dahlia* and *Fusarium oxysporum* (soil-borne fungal pathogens). Bacterial assemblages connected with plant roots contribute a vital role in subduing soil-borne pathogens, and multispecies probiotic associations could boost disease suppression efficiency. For example, Hu, et al. [30] reported that the addition of *Pseudomonas* consortia in *Solanum lycopersicum* rhizosphere microbiome reduced *Ralstonia solanacearum* concentration and lessened the disease incidence because of the meddling and increased resource competition with the pathogen. Similarly, an increase in the *Pseudomonas* consortia richness resulted in increased plant biomass and effective absorption of nutrients in *Solanum lycopersicum* plants.

Root-related microbes likewise produce communicating compounds ranging from antibiotics, organic acids, volatile signals, phytohormones, extracellular enzymes and quorum-sensing molecules (QSMs) (Figure 2) [31]. These compounds aid the relationship between plant roots and microbes associated with facilitating plant growth. For instance, N-acyl-L-homoserine lactones (quorum-sensing molecules) were reported by Ortiz-Castro [32] to influence the lateral root formation, root system architecture, primary root growth, and root hair development of *Arabidopsis thaliana* in their post-embryonic stage. Bacterial strains such as *Bacillus amyloliquefaciens* L3 use communicating molecules like volatile organic compounds to stimulate reactions in fungi and plants and generate induced systemic resistance (ISR) in plants, consequently the eliciting expression of defense genes that mitigate the negative effect of viruses, oomycetes, bacteria, and fungi on plants [33,34,35,36]. Root-resultant exudates, apart from aiding plant fitness and longevity, also benefit microorganisms that use them as a resource (carbon-rich products with other nutrients) that supports microbial multiplication [37,38,39].

Alongside different rhizodeposits produced in the rhizosphere, different hormones are also produced that aid plant-microbe communication cascades [18]. These hormones include abscisic acid, auxin, cytokinins, gibberellin, and peptide hormones that regulate plant growth and development. Several plant growth-promoting rhizobacteria have been reported to produce indole acetic acid in a chemically defined medium with tryptophan precursor [40,41]. Auxin production enhances seed germination, nutrient uptake, and root growth and development [18,42]. For instance, cytokinin was found to stimulate cell division, inhibit root elongation, and affect root hair development [43], while gibberellin was reported to alter many physiological and developmental processes in plants by promoting seed germination, stem elongation, flowering, and fruit setting in plants [44]. Gibberellin also facilitates cell-to-cell communication [18].

In the past, microbial ecologists used to face big challenges in investigating multifaceted microbial societies. However, today the tide has changed thanks to methodological advances like the high throughput deoxyribonucleic acid sequencing machinery that provides comprehensive information on the composition and structure of microbial groups [45,46,47]. With the availability of many sequence datasets from environmental samples, the focus now is to go beyond alpha and beta diversity and look more at the interactions between microbial taxa and their host [48,49]. To obtain a more profound understanding of plant–root-microbe interactions, new tools are being developed. For instance, exometabolomics have been developed to dissect cross-feeding between root microorganisms and plants when root exudates serve as the only carbon source for the cultivation of the rhizosphere microbes [50]. With this tool, it is now possible to know the main compound controlling plant-bacteria communications by comparing exometabolite datasets [51]. Another available tool is the synthetic microbial communities (SynComs) approach, which is used to expound and predict outputs caused by particular characteristics of bacterial consortia [52]. This SynComs approach was used by Lebeis, et al. [53], who revealed that the defense phytohormone salicylic acid modulates bacterial colonization of the roots of Arabidopsis. These available tools allow us to unknot the entangled networks of interactions of fundamental microbiomes or holobionts for efficiently using the root microbiome to intensify crops’ nutrient procurement and fight against biotic and abiotic stress [54].

## 3. Chemical Communication Mechanisms between Plants and Microbes and Their Application in Plant Growth and Yield

Microorganisms use processes like enzymes release, induction of systemic resistance in host plants, antibiosis, and rhizospheric competence to stimulate plant-microbe communications, and these processes originated from chemical signaling [55]. Distinct chemical signal molecules produced by different microbes influence biofilm growth, sporulation, motility, conjugation, virulence, antibiotic production, and symbiosis as well as alter the soil pH and the microbial community [56]. Most of these chemicals are used to induce tolerance against biotic and abiotic stress. Cho, et al. [57] reported that *Pseudomonas chlororaphis* O6 produced 2R, 3R-butanediol in the presence of jasmonic acid, ethylene, and salicylic acid signaling pathways, increasing the tolerance to drought in *Arabidopsis thaliana* by increasing the proportion of closed stomata. Another study revealed that under salt stress conditions, *Pseudomonas simiae* strain AU produced volatile organic compounds that provided salt tolerance in soybean plants by decreasing sodium ion and increasing the amount of potassium and phosphorus content [58]. Their investigation further revealed that proline and chlorophyll content increased in the plant roots exposed to volatile organic compounds released by *Pseudomonas simiae* strain AU [58]. Some chemicals produced by microbes have the ability to activate a chain of physiological alterations that stimulate plant growth [59]. del Carmen Orozco-Mosqueda, et al. [60] reported that the release of dimethylhexadecylamine by *Arthrobacter agilis* UMCV2 increased iron obtainability in *Medicago truncatula.* This became possible due to (i) the dimethylhexadecylamine released by *Arthrobacter agilis* UMCV2 stimulating acidification in the root biome. (ii) This then promoted proton extrusion under an iron deficit. (iii) Finally, it led to an increase in ferric reductase activity. Fungal colonization primes the chemical protection development in plants by increasing the levels of fatty acid derivatives, alkaloids, terpenoids, and phenylpropanoid polyamine conjugates in plants; these compounds are emitted to prevent pests [61,62]. The plant root-associated microbes in mitigating abiotic and biotic stress, nutrient acquisition, and growth promotion must be promoted to enhance sustainable agriculture [63,64].

### 3.1. Root Microbiome Role in Abiotic Stress Tolerance

Crops are often exposed to physical stresses such as soil salinization, submergence, extreme temperatures, nutrient imbalances, and drought, to mention a few [65]. The fact that these stresses will intensify in the coming years makes them a big concern as plant growth, yield, and productivity will be hindered. To overcome these abiotic stresses, plants must undergo adaptive modifications or solicit the help of beneficial microbes to live and promote plant function [46,66]. Root-associated microbes can stimulate growth and defend the host via many molecular machinery in abiotic stress circumstances (Table 1). Ribeiro and Cardoso [67] revealed that strains of *Bacillaceae*, *Enterobacteriaceae*, and *Pseudomonadaceae* isolated from the *Araucaria angustifolia* root were tremendous plant growth-promoting bacteria. Some of these bacterial strains are P-solubilizing microbes that help the crops tolerate drought, salt, and extreme temperature conditions through the production of numerous phytohormones, antioxidants, and exopolysaccharides; the production of 1-aminocyclopropane-1-carboxylate deaminase; the enrichment of nutrient uptake; the production of many volatile compounds; and the initiation of the buildup of osmolytes. They also help in the regulation of stress-responsive genes [68,69]. Under drought settings, Yuwono, et al. [70] found that osmotolerant rhizobacterial inoculated with rice increased root and shoot dry weight. It was also proven that under stress conditions, these isolates produced betaine, signifying that the drought tolerance was because of the increase in osmolyte. They also revealed that the rhizobacteria-plant interaction led to indole acetic acid production. An under-drought experiment conducted by Ruiz-Lozano, et al. [71] showed that co-inoculation of *Glomus mosseae* and *Bradyrhizobium japonicum* in drought-stressed soybean plants resulted in increased leghemoglobin content, acetylene reductase activity, and protein content by 25%, 112%, and 15%, respectively, compared with well-watered soybean plants and plants colonized by *Bradyrhizobium* alone.

Root-associated microbes also use other strategies to retain ion homeostasis in plants facing salt stress. For instance, bacterial exopolysaccharides fix Na^+^ and confine Na^+^ inflow into the roots. They produced volatile organic compounds (VOCs) during stress conditions so that VOCs can activate high-affinity K^+^ transporter (HKT1) reduction in roots and stimulate HKT1 in shoots, thereby restraining Na^+^ entrance into roots and easing shoot-to-root Na^+^ retransmission. The K^+^/Na^+^ ratio is increased by arbuscular mycorrhizal fungi by immensely improving Ca^2+^ and K^+^ absorption and eluding the movement of toxic Na^+^ under salty conditions. Furthermore, for effective water assimilation in saline-strained plants, roots’ closely associated microbes control the processes of genes encrypting the plasma membrane integral proteins to aquaporin activity [72,73,74]. Boosting the antioxidative systems in plants for ROS (reactive oxygen species), scavenging, and producing polyamines and proline are among the mechanisms employed by root-associated microbes in mitigating salt stress in plants. Bano and Fatima [75] induced salt stress conditions and co-applied *Pseudomonas* and *Rhizobium* at the seedling stage of maize. Their findings showed that under sodium chloride conditions alone, a harmful effect on maize growth and development was observed. Furthermore, improved sodium chloride tolerance of maize upon co-inoculation with *Pseudomonas* and *Rhizobium* is linked with reduced electrolyte leakage, increased proline production, and conservation of leaf water contents. The improvement of nutrient uptake to boost plant survival under salt conditions is another mechanism employed by root-associated microbes. For instance, the introduction of *Bacillus aquimaris* to wheat plants resulted in a substantial rise in phosphorus, nitrogen, and potassium in wheat leaves (Upadhyay and Singh 2015). The root-associated microbe can also aid plants in withstanding high- or low-temperature conditions, either by increasing or decreasing anthocyanin, proline, and sugar contents. Barka et al., [76] reported that under low temperatures, grapevine plant bacterized with *Burkholderia phytofirmans* strain PsJN increased physiological activity and grapevine growth through a substantial increase in proline, starch deposition, carbohydrates, and phenol contents compared with the control.

**Table 1:** Root-associated microbes and their mitigation of abiotic stresses confronting plants.

Stress Type	Root Associated Microbes	Plant Host	Inoculated with	Activities	The Effect on Plant	Reference
Drought	*Enterobacter*, *Bacillus*, *Moraxella* and *Pseudomonas*	*Acacia arabica*	*Triticum aestivum* L.	Indole-3-carboxylic acid, indole-3-lactic acid, and indole-3-acetic acid production	Improved shoot length, tillers, and number of spikelets and increased spike length and seed weight of *Triticum aestivum* L.	[77]
Salt	*Halomonas* and one *Bacillus*	*Salicornia rubra*, *Sarcocornia utahensis*, and *Allenrolfea occidentalis*	Alfalfa	-	Increased total biomass of alfalfa and improved root length by 2.6 and 1.5 fold in *Halomonas* and *Bacillus* inoculated plants, respectively, compared with the uninoculated alfalfa.	[78]
salt or drought	*Bacillus amyloliquefaciens* SB-9	Grapevine	Grapevine plantlet	melatonin secretion, 5-hydroxytryptophan, serotonin, and *N*-acetylserotonin	Lessened the antagonistic effects of salt- and drought-induced stress by decreasing the secretion of malondialdehyde, O_2_^-^, and H_2_O_2_ (reactive oxygen species) in roots.	[79]
Heavy metal stress	*Phialocephala fortinii*, *Rhizodermea veluwensis*, and *Rhizoscyphus* sp	*Clethra barbinervis*	*Clethra barbinervis* seedling	Siderophores	Improved K absorption in shoots and decreased the concentrations of Cd, Zn, Pb, Cu, and Ni in roots.	[80]
Heavy metal	*Penicillium ruqueforti* Thom	*Solanum surattense* Burm	Wheat seedling	Indole-3-acetic acid	Led to low concentrations of heavy metals in the root and shoot. Increased nutrient uptake and higher plant growth.	[81]
Heat	*Thermomyces* sp.	*Cullen plicata*	Cucumber	Increase in antioxidant enzyme activities, soluble proteins, flavonoids, saponins, and total sugars.	Maintained the optimal quantum efficiency of photosystem II, water use efficiency, and photosynthesis rate and increased the root length, induced accumulation of saponins, total sugars, soluble proteins, flavonoids, and antioxidant enzyme activities.	[82]
High temperature, salinity, and glyphosate pollution	*Ochrobactrum cytisi* strain IPA7.2	*Solanum tuberosum* L.	*Solanum tuberosum* L.	Indole-3-acetic acid and type II 5-enolpyruvylshikimate-3-phosphate synthase	Improved the mitotic index of root meristem cells, the number of roots, the number of leaves and the length of shoots.	[83]
Flood	*Klebsiella variicola* AY13	Soybean	Soybean	Indole acetic acid production	Plants growth improved with enriched chlorophyll content and quantum efficiency of chlorophyll fluorescence.	[84]

### 3.2. Root Microbiome Role in Nutrient Acquisition

Most micronutrients and macronutrients important for plant growth are available in the soil in insoluble forms. Plants devise several mechanisms for the acquisition of these nutrients in the soil. The plant root microbiome enhances the uptake of major micronutrients by mineralizing or solubilizing them and ensuring their bioavailability through acidification [85,86], secretion of hydrolytic enzymes such as phytase or phosphatase, excretion of proton, and production of siderophore [87]. Endophytes, rhizospheric microbiomes, and arbuscular mycorrhizal fungi (AMF) help the plant in the acquisition of nutrients from the soil through the solubilization of nutrients such as sulfur (S), potassium (K), calcium (Ca), iron (Fe), zinc (Zn), and phosphorus (P) [88,89,90]. Some notable root microbiome genera associated with maize, wheat rice, and legumes, such as *Streptomyces*, *Pantoea*, *Citrobacter*, *Azospirillum*, *Bacillus*, *Herbaspirillum*, *Achromobacter*, *Gluconacetobacter*, *Burkholderia*, *Chryseobacterium*, *Bacillus*, *Klebsiella*, *Azotobacter,* and *Pantoea,* have been reported to enhance plant development and growth via the uptake of micronutrients and stimulate the development of plant roots [91,92,93,94]. Siderophores secreted by endophytes aid plants’ iron uptake from the soil; this is because iron cannot directly penetrate the plant cell even through transporters [95]. Root endophytes, such as *Azoarcus*, *Herbaspirillum*, *Acetobacter,* and diazotrophicus, have been reported to be active in nitrogen fixation. Some diazotrophic endophytic microbial communities, such as *Bacillus*, *Gammaproteobacteria*, and *Actinobacteria,* have been largely reported as atmospheric nitrogen fixers in rice [95,96,97]. Rhizobia, most importantly, *Burkholderiales*, form root nodules with legumes, which convert atmospheric nitrogen into ammonia, which is readily available to the plant, while the plant, in return, produces carbon compounds [98,99]. Some root microbiomes such as *Brevibacillus*, *Kineococcus*, *Microbacterium*, *Rhizobium*, *Burkholderia*, *Nocardia*, *Bacillus*, *Rhodococcus*, *Methylobacterium*, *Mesorhizobium*, and *Paenibacillus* associated with Eucalyptus plant have been reported to be involved in the fixing of nitrogen [95,100]. A summary of other studies on the nutrient acquisition attributes of some plant root microbiomes is presented in Table 2.

### 3.3. Root Microbiome Role in Disease Suppression/Biocontrol

Insects and pathogens attack plants and retard their yield, growth, and health. However, plant root microbiomes have been reported to be a reservoir of many bioactive metabolites that can protect and enhance plant resistance against attacks from pathogens and pests [95]. Phyllospheric microorganisms isolated from different plants showed that an abundance of Firmicutes is capable of secreting volatile organic compounds active in the protection of crops from several fungal and bacterial pathogens/diseases [119]. The plant root microbiome protects the plant through induced systemic resistance (ISR) or antibiosis from insects, pathogens, and herbivores. Siderophores, antibiotics, salicylic acid, N-acyl homoserine lactones, lipopolysaccharide, jasmonic acid, and flagella secreted by endophytic bacteria have been reported to be capable of inducing systemic resistance in plants [120]. In addition, endophytic fungi, majorly of the phyla *Glomeromycota*, *Basidiomycota*, *Ascomycota*, and *Zygomycota*, are capable of secreting inhibitory compounds, some of which are terpenoids, polyketones, phenols, chlorinated compound, alkaloids, peptides, steroids, and flavonoids, which aid the protection of plants from insects, pathogens, and herbivores [121]. Actinomycetes have also been widely studied due to their ability to secrete notable antimicrobial compounds active against plant pathogens. *Streptomyces* spp. secretes many antimicrobial compounds such as indolo-sesquiterpene antimicrobial compounds, munumbicins, coronamycin, and kakadumycins [95,122,123]. Studies have also revealed that siderophore can induce ISR in plants and enhance biocontrol activities. For example, strains of endophytic methylobacterium successfully suppressed *Xylella fastidiosa* (a pathogen responsible for chlorosis in citrus trees) via siderophore production [63]. Rhizobiomes such as Actinobacteria, Proteobacteria, and Firmicutes have been linked with the inhibition of *Rhizoctonia solani*, which commonly attacks sugar beet [95], while *Gammaproteobacteria* have also successfully inhibited the disease via non-ribosomal peptide synthesis (NRPS) [124]. A high abundance of bacteria such as *Streptomyces*, *Bacillus*, *Paenibacillus*, and *Rhizobium* in the root microbial community of cucumber was cultivated and monitored in suppressive soil [125]. A summary of similar studies on the biocontrol attributes of some plant root microbiomes is presented in Table 3.

## 4. Conclusions and Future Prospects

It is evident that plant-microbe signaling cascades are essential regulators of plant development and growth, and these signal molecules can alter the morphology and physiology of the host plant. Plants develop complex interactions and communicate with various microbes in their rhizosphere through different signals that affect plant growth and modulate the plant-specific core root microbiome. These signals, secreted by micro- and macro-symbionts, can enhance root development, increase nutrient and water uptake, and promote tolerance to biotic and abiotic stresses. As a result of the roles that plant growth-promoting organisms played in improving plant growth and yield, the role of plant-microbe signals in sustainable agriculture and the recovery of marginal lands cannot be overemphasized. It is, therefore, important to focus future research on the understanding of intra- and inter-communication that can lead to the identification of more signal molecules and similarly improve plant growth and development. There is a need to develop efficient technologies for isolating and identifying signal compounds useful for sustainable development.

## Figures and Tables

**Table 2:** Nutrient acquisition attributes of notable plant root microbiomes.

Root Microbiomes	Host Plant	Phosphorus (P)	Potassium (K)	Nitrogen Fixers (N_2_F)	Siderophore (Sid)	Zinc (Zn)	References
*B. amyloliquefacien*	Rice	+	+	+	+	+	[101]
*A. sulfonivorans*	Wheat	−	−	−	+	+	[102]
*A. amazonense*	Sugarcane	−	−	+	−	−	[103]
*B. megaterium*	Soybean	+	−	+	+	−	[104]
*P. agglomerans*	Rice	+	−	+	−	−	[101]
*P. putida*	Soybean	−	−	+	+	−	[105]
*B. silvatlantica*	Sugarcane	−	−	+	−	−	[106]
*B. aryabhattai*	Soybean	−	−	−	−	+	[107]
*K. pneumoniae*	Rice	−	−	−	+	−	[108]
*B. tropica*	Sugarcane	−	−	+	−	−	[109]
*P. putida*	Rice	+	−	−	−	−	[110]
*P. dispersa*	Wheat	−	−	−	+	+	[101]
*B. vietnamiensis*	Rice	−	−	+	−	−	[111]
*R. leguminosarum*	Beans	+	−	−	+	+	[112]
*B. licheniformis*	Chickpea	+	−	−	−	−	[113]
*B. subtilis*	Soybean	−	−	+	+	−	[114]
*P. polymyxa*	Maize	−	−	+	−	−	[115]
*P. thivervalensis*	Maize	−	−	−	+	−	[116]
*E. asburiae*	Maize	−	−	−	+	−	[116]
*R. endophyticum*	Beans	+	−	−	−	−	[117]
*R. irregularis*	Tomato	+	−	−	−	−	[118]

+ Active, − Inactive.

**Table 3:** Biocontrol activities of some plant root microbiomes.

Root Microbiomes	Host Plant	Pathogens Active against	Activities and Metabolites Secreted/Induced	References
*Pseudomonas* sp., *Pantoea* sp.	Grapevine	*A. tumefaciens*, *A. vitis*	-	[126]
*A. calcoaceticus*	Soybean	*P. sojae* 01	Siderophore and indole acetic acid	[105]
*Bacillus* sp.	Soybean	*C. truncatum*, *R. solani*, *F oxysporum*, *S. rolfsii*, *A.* *alternata*, and *M. phaseolina*	Siderophore and Hydrogen cyanide.	[127]
*B. subtilis*	Rice	*R. solani*, *F. verticelloides*, and *S. rolfsii*	Lipopeptides	[128]
*B. gladioli* 3A12	Maize	*S. homoeocarpa*	-	[129]
*P. fluorescens* 63–28	Pea	*P. ultimum* and *F. oxysporum* f. sp. pisi	Induced peroxidase, polyphenoloxisae, Superoxide dismutase and phenylalanine amonialyase.	[130]
*P. aeruginosa* FTR	Maize	*F. oxysporium*, *P. aphanidermatum*, *Alternaria* sp., *R solani*, *M. phaseolina*, *Alternaria* sp. and *S. rolfii*,	-	[116]
*Glomus etunicatum*	Wheat	*G. graminis*	Isozyme	[131]
*B. velezensis* CB3	Citrus	*P. digitatum*	-	[132]
*G. versiforme* and *T harzianum*	Cowpea	*E. flexuosa*	-	[133]
*B. velezensis*	Maize	*T. funiculosus*, *P.* *oxalicum*, and *F. verticillioides*	Lipopeptide	[134]
*R. leguminosarum* RPN5	Beans	*M. phaseolina*, *F.* *oxysporum*, *S. sclerotiorum* and *F. solani*.	-	[112]
*Serratia* (B17B), *Enterobacter* (E), and *Bacillus* (IMC8, Y, Ps, Psl, and Prt)	Papaya and Bean	*P. capsici*	-	[135]
*Acremonium* sp., *Leptosphaeria* sp., *T. flavus*, and *P. simplicissimum.*	Cotton	*V. dahliae* strain Vd080	-	[117]
*Bacillus* sp.	Millet	*R. solani*, *S. rolfsii*, and *F. solani*	Antimicrobial peptides	[136]
*B. subtilis*	Rice	*M. oryzae*	Enhanced activity of peroxidase, polyphenol oxidase and superoxide dismutase	[137]
*Pseudomonas* sp.	Wheat	*F. graminearum*	-	[138]
*B. subtilis* EB-28	Tomato	*B. cinerea*	-	[139]
*F. mosseae*	Wheat	*X. translucens*	-	[140]
*R. irregularis*	Tomato	*A. solani*	-	[118]
*F. mosseae*	Wheat	*B. graminis*	-	[141]
*F. mosseae* and *P. fluorescens*	Wheat	*G. graminis*	-	[142]

## Data Availability

Not applicable.
